# Fasting-Based Dietary Interventions in Cancer Patients and Survivors: A Scoping Review

**DOI:** 10.3390/nu18071035

**Published:** 2026-03-25

**Authors:** Kuang-Yi Wen, Julianne Freedman, Abenezer Tafese, William Kelly, Nicole Simone

**Affiliations:** 1 Department of Medical Oncology, Thomas Jefferson University, 834 Chestnut Street, Suite 320, Philadelphia, PA 19107, USA; julianne.freedman@jefferson.edu (J.F.);; 2 College of Population Health, Thomas Jefferson University, 901 Walnut Street, Philadelphia, PA 19107, USA; 3 Department of Radiation Oncology, Thomas Jefferson University, 111 South 11th Street, Philadelphia, PA 19107, USA

**Keywords:** time-restricted eating, cancer, intermittent fasting, survivorship

## Abstract

**Background**: Fasting-based interventions are increasingly investigated as adjuncts to cancer treatment for the potential to reduce therapy-related toxicities, improve metabolic health, and enhance quality of life. However, clinical evidence regarding their efficacy, tolerability, and acceptability remains limited and fragmented. This scoping review aimed to systematically map the current evidence on fasting-based interventions in cancer patients and survivors. **Methods**: A literature search was conducted in PubMed, Scopus, Web of Science, and CINAHL up to 10 June 2025. Eligible interventional studies included cancer patients or survivors and evaluated fasting-based interventions, such as time-restricted eating, intermittent fasting, short-term fasting, or fasting-mimicking diets. Studies were categorized by fasting types and outcomes like fatigue, treatment toxicity, metabolic and hematologic parameters, weight, quality of life, adherence, acceptability, illness perception, and adverse events were assessed. **Result**: Twenty interventional studies of FMD, TRE, STF, IF, or fasting combined with altered dietary approaches conducted across 10 countries were included, comprising a total of 871 participants. Participant ages ranged from 28 to 75 years. Overall, 9 of 20 studies exclusively enrolled breast cancer patients or survivors, and chemotherapy was the most common treatment context in 11 studies. Five of six studies reported reductions in fatigue. Among the five studies assessing quality of life, one demonstrated improvement, three reported no change, and one yielded mixed results. Six of eight studies reported reductions in chemotherapy-related toxicity, and weight loss was observed in 10 of 12 studies. Reductions in IGF-1 and insulin levels were reported in six of seven and four of five studies, respectively. Hematologic changes were noted in six studies, and only one study assessed illness perceptions, reporting positive findings. Fasting-related adverse events, reported in nine studies, were generally mild and transient. High adherence and acceptability were observed across studies; however, findings were heterogeneous across intervention types and were largely derived from small or moderate-strength studies. A descriptive quality metric assessment indicated that most studies were of moderate methodological strength. More intensive fasting protocols, such as FMD and STF, appeared to demonstrate more consistent metabolic effects, whereas TRE showed higher adherence but more variable clinical outcomes. **Conclusions**: Fasting-based interventions have the potential to be feasible and well tolerated among cancer patients and survivors, with early evidence suggesting benefits in reducing fatigue, minimizing treatment-related toxicities, and favorable metabolic effects. Large, well-designed trials including diverse cancer populations are needed to confirm long-term outcomes and guide clinical integration.

## 1. Introduction

Cancer is the second leading cause of death from non-communicable diseases (NCDs), accounting for approximately 23% of all NCD-related mortality, second only to cardiovascular disease [1]. In 2022, an estimated 19.98 million new cases and 9.74 million cancer-related deaths were reported globally, with projections indicating that these figures will exceed 35 million new cases and 18.5 million deaths annually by 2050 [2]. Nearly half of cancer deaths worldwide are attributed to modifiable factors, making them potentially preventable [3]. Diet is one of the most important modifiable factors linked to cancer incidence and mortality. In 2019, approximately 6% of global cancer deaths and 5.5% of cancer-related disability-adjusted life years (DALYs) were attributable to dietary risk factors, including low intake of whole grains, fruits and vegetables, and high consumption of red and processed meats [4]. Numerous studies have examined how unhealthy diets contribute to cancer development, leading to widely accepted dietary guidelines and recommendations for cancer prevention [5].

While much of the existing literature has emphasized what constitutes a nutritious diet, emerging studies have begun to explore the significance of the timing of food intake and the role of fasting-based interventions in cancer care. Observational studies have shown that late-night eating and shorter overnight fasting durations are associated with increased risks of cancer incidence and recurrence, supporting the rationale for conducting clinical trials that explore the role of fasting-based treatment regimens [6,7,8]. One of the most widely studied interventions is intermittent fasting (IF), defined as alternating periods of eating and fasting. IF has gained attention for its ability to improve cardiometabolic health and reduce systemic inflammation [9,10]. IF is an umbrella that includes many different fasting regimens. Examples include (1) a fasting-mimicking diet (FMD), a structured, low-calorie diet typically followed for 5 days with repetition of the diet every few weeks [11]; (2) time-restricted eating (TRE), which limits daily caloric intake to a consistent 4–12 h window aligned with circadian rhythms [12]; and (3) short-term fasting (STF) or modified short-term fasting (mSTF), which typically involves water-only fasting periods lasting more than 24 h and up to 72 h [13] (Table 1).

The mechanisms underlying IF and other fasting-based interventions in oncology are not yet fully understood. However, preclinical and early-phase clinical studies suggest several plausible pathways. Fasting has been shown to reduce circulating insulin and insulin-like growth factor-1 (IGF-1), which in turn inhibits the PI3K/Akt/mTOR signaling axis and activates autophagy. These processes are associated with enhanced DNA repair, tumor suppression and differential stress resistance (DSR) [14,15,16]. DSR is the phenomenon in which starved or nutrient-scarce conditions render cancer cells more susceptible to cancer therapies while protecting normal cells from high-dose chemotherapy or oxidative stress [17]. Fasting may also decrease pro-inflammatory cytokines and angiogenic mediators such as vascular endothelial growth factor (VEGF), while restoring circadian regulation of cell cycle and metabolic genes [15,18,19]. Together, these biological effects may contribute to improved treatment response, reduced toxicity, greater treatment tolerance, and enhanced quality of life in cancer patients [20].

Despite growing interest, evidence on fasting-based dietary interventions in oncology care remains limited to early-phase pilot trials conducted across heterogeneous cancer populations. Existing studies vary widely in intervention protocols, timing relative to treatment, and outcome measures. Moreover, there is a lack of comprehensive data on key clinical endpoints such as treatment-related toxicities, fatigue, metabolic and hematologic parameters, adherence, and survivorship. This scoping review addresses these gaps by systematically mapping clinical evidence on fasting-based dietary interventions among cancer patients and survivors. It aims to characterize the different fasting protocols, feasibility, and outcomes of these interventions; to highlight emerging trends; and to inform future research directions.

## 2. Materials and Methods

### 2.1. Study Design

This scoping review was conducted in accordance with the Preferred Reporting Items for Systematic Reviews and Meta-Analyses extension for Scoping Reviews (PRISMA-ScR) guidelines. The completed PRISMA-ScR checklist is provided in Supplementary File S1.

### 2.2. Eligibility Criteria

The eligibility criteria were established using the population, intervention, comparator, and outcome (PICO) framework. Studies were included if they met the following characteristics: (1) populations: individuals with cancer, including any type or stage, and cancer survivors; (2) intervention: time-restricted eating/diet/feeding, 16:8 diet, intermittent fasting, fasting-mimicking diet; (3) comparators: control group or standard of care, pre–post assessment, feasibility and safety; (4) outcomes: data on at least one of the following: cancer fatigue, treatment-related toxicity, metabolic parameters, hematologic parameters, adverse effects, weight loss, cancer recurrence risk, intervention adherence, acceptability and illness perceptions. Studies were excluded if they involved healthy individuals or those without cancer; examined non-fasting dietary interventions or religious fasting; focused on cancer prevention; investigated other metabolic diseases; or were non-interventional in design.

### 2.3. Search Strategy

A comprehensive literature search for peer-reviewed studies was conducted in PubMed, Scopus, Web of Science, and CINAHL from database inception through 10 June 2025. The search strategy combined medical subject headings (MeSH) and free-text terms related to intermittent fasting and cancer. No filters or restrictions were applied during the initial search. Search strings included (‘Neoplasms” [MeSH] OR cancer* OR tumor* OR malignancy OR Survivors) AND (“Fasting” [MeSH] OR ‘Time-Restricted Eating” [MeSH] OR fasting OR “intermittent fasting” OR “fasting mimicking diet” OR “16:8 diet”). Additionally, the reference lists of included articles were reviewed to identify any additional relevant studies.

### 2.4. Selection Process

All identified articles were screened against predefined eligibility criteria. Two reviewers conducted the initial screening of titles and abstracts. Subsequently, full-text articles of potentially relevant studies were retrieved and independently assessed by two reviewers to determine final inclusion. No discrepancies were identified during the full-text review; therefore, adjudication by a third reviewer was not required. 

### 2.5. Data Extraction

Data from all eligible studies were independently extracted by two reviewers using an adapted template in Microsoft Excel. Extracted information included author, year, study population characteristics (such as sample size, age, race and ethnicity, cancer type, treatment trajectory); study design (including type, length of intervention, modality, primary endpoint); reported outcomes (such as fatigue, quality of life, toxicities, weight loss, cancer recurrence risk, hematologic parameters, metabolic parameters, acceptability, illness perception, adherence to intervention, adverse events); and endpoint follow-up with lessons learned. The data were then refined for further analysis. 

### 2.6. Quality Assessment

In accordance with the PRISMA-ScR guidelines, this review does not include a formal critical appraisal of individual studies [21]. To provide additional context regarding the relative strength of the evidence, a descriptive scoring framework was developed evaluating five methodological domains: study design, sample size, intervention characterization and protocol clarity; outcome measurement and reporting quality; and analytical rigor [22]. Each domain was scored on a 0–2 scale, yielding a maximum score of 10 points per study. The total score for evidence strength is categorized as low strength (0–3), moderate strength (4–7) and high strength (8–10). This framework was applied to characterize the robustness of the existing evidence base and was not used to determine study inclusion. The domain scoring is outlined below (Table 2, Table 3, Table 4, Table 5 and Table 6).

### 2.7. Data Synthesis

Extracted data were synthesized descriptively to map the existing evidence on the impact of fasting interventions among individuals with cancer or survivors. Studies were first summarized based on key characteristics, including participant demographics, study design, geographic location, treatment trajectory, intervention design, and outcomes assessed. Findings were then organized thematically by fasting intervention type and then outcome domain, including changes in cancer-related fatigue, quality of life (QOL), treatment-related toxicities, weight, hematologic and metabolic parameters, as well as intervention acceptability, adherence, illness perceptions, and reported adverse events.

## 3. Results

### 3.1. Study Selection

**Figure 1:**
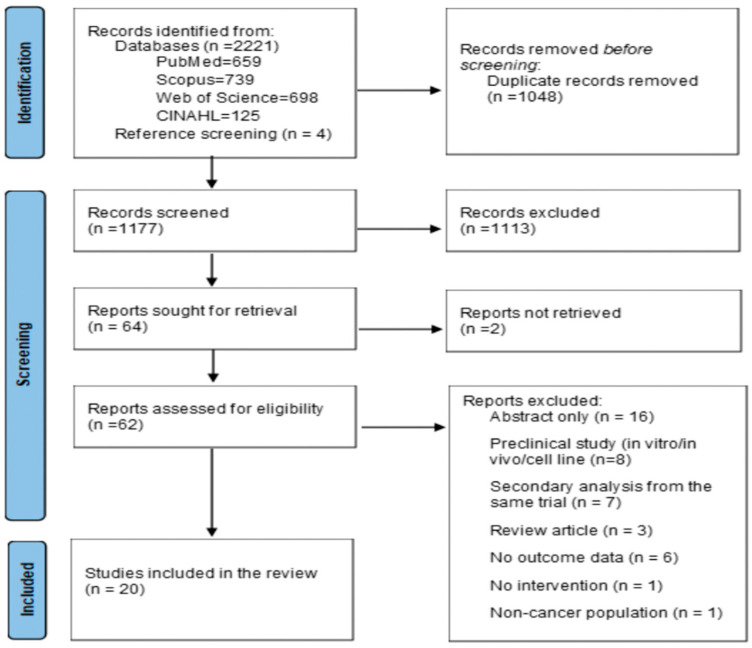
PRISMA flow diagram.

A total of 2225 records were identified: 2221 through database searches (PubMed = 659, Scopus = 739, Web of Science = 698, and CINAHL = 125), and four through reference screening. After removing 1048 duplicates, 1177 records remained for title and abstract screening. Of these, 1113 were excluded, and 64 reports were sought for retrieval. Two reports could not be retrieved, resulting in 62 reports assessed for eligibility. Among these, 42 were excluded for the following reasons: conference abstract only, with no full report available (n = 16); preclinical study (in vitro, in vivo, or cell line) (n = 8); secondary analysis from the same trial (n = 7); review article (n = 3); no outcome data (n = 6); no intervention (n = 1); and non-cancer population (n = 1). Ultimately, 20 studies met the inclusion criteria and were included in the final analysis (Figure 1). 

### 3.2. Strength of Evidence

Of the twenty studies reviewed, two were rated as high strength, reflecting randomized controlled trials (RCTs) with robust methodology, protocol and analytical rigor [17,23]. Seventeen studies were rated as moderate strength due to a single-arm, pilot, or open-label design with a small sample size but moderate–strong intervention characterization, outcome reporting and analytical rigor [24,25,26,27,28,29,30,31,32,33,34,35,36,37,38,39,40]. One RCT was rated as moderate strength due to a small sample size (n = 20) [27]. One study was rated as low strength due to a prospective non-randomized design with a small sample size (n = 21) and low strength of outcome reporting and measurement quality [41]. Overall, these findings indicate that the current evidence base is predominantly composed of moderate-strength studies, with relatively few high-strength trials, highlighting the need for larger, well-designed randomized studies to strengthen the evidence in this field (Table 7).

### 3.3. Study Characteristics

Individual study characteristics, including key elements of the intervention, study design, participant characteristics, disease site, treatment trajectory and outcomes, are outlined below according to FMD, TRE, STF/mSTF, IF and fasting + altered diet regimens. Across the 20 included studies, there were 871 participants in total, with an average sample size of 44. The study sample sizes ranged from 13 [24] to 129 participants [30]. Most participants were aged between their mid-40s to mid-50s, with the lowest reported age being 28 [26] and highest being 75 [25]. The gender makeup of the study samples was reported across seven studies [25,31,32,33,34,35,38], with a predominance of females in five studies [25,31,32,33,34] and one study enrolling males exclusively [35]. The designs of the 20 included studies consisted of three RCTS [17,23,27], seven various randomized studies (i.e., pilot, feasibility or prospective) [24,26,28,30,37,38,39], four non-randomized designs [25,29,40,41], and six single-arm designs [31,32,33,34,35,36]. Of the total extracted studies, five were FMD interventions [17,32,34,35,39], four were TRE interventions [33,36,38,41], three were STF/mSTF interventions [24,25,27], two were IF interventions [23,37], and six were fasting + altered diet interventions [26,28,29,30,31,40]. These studies were conducted in the United States [25,27,31,33,38], Germany [26,28,29,37], Italy [32,34,39], the Netherlands [24,30], Canada [36], Chile [41], Iran [17], Pakistan [40], Ireland [35] and Egypt [23]. The populations assessed included patients with breast cancer exclusively [17,23,24,30,37,39,40,41], cancer survivors [33,36,38], brain cancer [28,31], prostate cancer [35], gynecological and breast cancer [25,26,27,29], and mixed cancer types, such as skin, gastrointestinal and lung [32,34]. The most common treatment in the included studies was neoadjuvant, adjuvant, anthracycline-based, or platinum-based chemotherapy [17,23,24,25,26,27,29,30,31,36,39,40] followed by radiotherapy with curative intent [28,37,41] and mixed treatment modalities (i.e., chemotherapy, radiotherapy and surgery) [32,33,34].

### 3.4. FMD Interventions

Of the five studies investigating FMD interventions, three reported on body weight or body mass, with findings indicating a reduction in weight or body mass index (BMI) [32,35,39]. Valdemarin et al. (2021) [32] conducted a single-arm (n = 90) 6-month, 5-day FMD cycle study of patients within several disease sites undergoing active anti-cancer treatment, resulting in an average weight loss of 2–2.5kg. Similarly, another single-arm study (n = 35) investigating a 3-month, 3-day FMD cycle in prostate cancer patients found an average 3.79kg weight loss [35]. Ligorio et al. (2025) [39] conducted a pilot randomized trial, finding that obese or overweight breast cancer patients undergoing neoadjuvant chemotherapy and completing a 5-day FMD cycle for 5 months (n = 13) had reduced their BMI score by 11.3% compared to the control group (n = 17) undergoing the same FMD cycle plus the administration of Metformin (3.7%).

All five studies assessed the impact of an FMD on metabolic parameters such as IGF-1, cholesterol and blood glucose, with all studies reporting a clinically significant reduction in IGF-1 levels [17,32,34,35,39]. Bahrami et al. (2024) [17] conducted an RCT in HER2-negative breast cancer patients completing a 4-day FMD for 5 months (n = 22) compared to a routine diet group (n = 22). The study found that IGF-1 levels were lower in the fasting group (172 ng/mL) than in the control group (181 ng/mL) after cycle 8 of chemotherapy [17]. IGF-1 levels were reduced at the end of the 5-day 6-month FMD period in a study conducted by Valdemarin et al. (2021) [32], and another study with an 8-month 5-day FMD for patients with various types of cancer on active anti-tumor treatment (n = 101) found similar IGF-1 reductions [34]. One study on prostate cancer patients found that the reduction of total cholesterol in participants with baseline levels under 5.2 mmol/L was clinically significant [35]. A study on breast cancer patients completing a 5-day FMD for 5 months found that the reduction in blood glucose levels was comparable in both the FMD group and FMD + Metformin group [39].

Adherence to the assigned intervention was a primary outcome for three of the five studies, with high adherence being reported for all. One study found 90% of participants completed one FMD cycle [32], while another found that 99% completed one FMD cycle in a 5-day FMD for 8 months while on different anti-tumor treatments [34]. Ligorio et al, (2025) [39] reported that 63.3% of participants were fully compliant with every cycle, but 88.9% completed every cycle with various deviations. Hematological parameters were an outcome assessed by two of the five studies, with reported reductions in low-density CD15+ granulocytes and total monocytes by day 5 of the FMD [34] and another study finding increases in erythrocyte and neutrophil count by the end of the FMD period [17].

Three studies investigated adverse events (AEs) attributable to the FMD intervention. A study of a 5-day FMD cycle for 5 months found 3% of the FMD group to have severe AEs attributable to the intervention compared to the 6.7% of the FMD + Metformin group experiencing severe AEs [39]. Treatment toxicity was assessed in one study of neoadjuvant chemotherapy for HER2-negative breast cancer patients undergoing a 4-day FMD cycle every 21 days for 5 months [17]. Findings indicate that grade I–III toxicities were the most observed, with the occurrence of grade III vomiting and neutropenia significantly higher in the control group than the FMD group (13.6% vs. 54.5%) (22.7% vs. 45.5%) [17]

In sum, across FMD studies, reductions in metabolic markers such as IGF-1 and improvements in treatment-related outcomes were generally observed; however, these findings were primarily derived from small pilot or feasibility trials. The magnitude and consistency of effects varied by intervention duration and number of cycles, and higher-strength studies demonstrated more modest or heterogeneous results, highlighting variability in intervention impact [17,32,34,35,39] [Table 8].

### 3.5. TRE Interventions

One study of 14-day, 14:10 TRE found a decrease in body weight by an average of 1.1–1.3 lbs by the end of the 14 days [33]. Another study of a 10 h eating window but with a 12-week duration found a slight increase in body weight in the TRE group and a slight decrease in body weight in the control group [38]. With a bigger fasting window of 16:8 TRE, one study found a reduction in BMI by 1 kg after 8 weeks [36]. For breast cancer patients undergoing radiotherapy treatment, Vega et al. (2024) [41] reported a significant weight loss of 6.84 kg in the TRE group after completion of a 12-week 16:8 TRE beginning 2 weeks before radiotherapy. All four studies investigated adherence to TRE. Kleckner et al. (2022) [33] found 90.1% of patients adhered to all 14 TRE days, and another study found that there was a median adherence of 98% for an 8-week 16:8 TRE [36]. Kleckner et al. (2025) [38] found that 90% of participants adhered to the 10 h eating window at week 6 and 100% adhered to the window at week 12. The last study reported 100% adherence to the 16:8 TRE for 12 weeks before the start of radiotherapy [41]. Three of the studies looked at the feasibility of TRE for cancer survivors with Kleckner et al. (2022) [33] reporting participants planning to continue 14:10 TRE with modifications and would recommend the diet to their peers. The other study found that 73% of the participants would continue a 16:8 TRE for 1 month post-intervention [36]. Vega et al. (2024) [41] looked at the loss of 5% of body weight from baseline as an indicator of feasibility due to true adherence. Compared to the calorie-restriction group (n = 16), the TRE group (n = 5) lost 5% of their body weight from baseline, meeting the feasibility endpoint [41]. Additionally, two studies assessed changes in cancer fatigue post-intervention with a 12-week, 10 h eating window leading to clinically meaningful improvements in functional assessment of chronic illness therapy fatigue (FACIT-F) scores (4.1 ± 5.7 vs. 0.0 ± 5.5; effect size = 0.70) [38] and a 2-week 14:10 TRE leading to improved FACIT-F by 5.3 ± 8.1 from baseline [33].

In sum, compared to FMD and STF protocols, TRE interventions demonstrated consistently high adherence rates but more modest and less consistent effects on metabolic and clinical outcomes. These findings should be interpreted in the context of the study design, as many TRE studies were small to moderate in sample size and varied in methodological rigor, with fewer high-strength randomized trials evaluating clinical endpoints. This suggests that while TRE may be more feasible and sustainable, its biological impact on oncological populations may be less pronounced or may require longer-term evaluation and larger, well-designed trials to fully characterize its effects [33,36,38,41] [Table 9].

### 3.6. STF or mSTF Interventions

All three studies implementing STF or mSTF interventions reported findings related to treatment toxicity outcomes after implementation. de Groot et al. (2015) [24] conducted a 4-month STF study examining the effects of a 24 h fast before and after chemotherapy for six cycles in women with stage 2 and 3 HER2-negative breast cancer (n = 7) compared to women following healthy nutrition guidelines (n = 6). Grade I and II toxicities were most observed in both groups, with the fasting group (71%) and control group (100%) reporting fatigue [24]. Another 4-month study of a 24 h fast before and after chemotherapy for six cycles in women with ovarian, uterine and cervical cancer (n = 10) compared to women consuming a balanced normo-caloric (NC) diet (n = 10) found that only grade I and II toxicities were reported in the fasting group [27]. Altogether, fewer grade II, III and IV toxicities were reported in the fasting group (8.3%) than the control (11.7%) [27]. Dorff et al. (2016) [25] conducted a non-randomized trial in patients with urothelial, uterine, cervical and breast cancer, receiving a dose escalation of fasting before and after two planned cycles of chemotherapy, starting at 24 h, escalating to 48 h and then 72 h. Grade I and II toxicities, such as nausea and vomiting, were reported in all cohorts with 100% reporting nausea and 83% vomiting in the 24 h cohort [25].

All three studies investigated changes in metabolic profile such as IGF-1 and insulin. A 4-month study of a 24 h fasting period before and after chemotherapy found a decrease in IGF-1 levels from baseline to the day of chemotherapy but found no statistically significant differences between the fasting and healthy eating group [24]. Dorff et al. (2016) [25] found that IGF-1 levels decreased in the 24 h, 48 h and 72 h fasting groups from baseline to the second and final cycle of chemotherapy, with the 48 h group having a 33% decrease. However, insulin levels decreased in each cohort after the first fast with the 24 h and 72 h groups having the highest median change (56% and 42%) [25]. Hematologic profile changes were investigated by two studies. Riedinger et al. (2020) found no significant differences in hemoglobin or neutrophil count between the fasting group and NC diet group, but found positive trends toward improved hematologic profiles in the fasting group [27]. The erythrocyte counts in the fasting group of stage 2 and 3 HER2-negative breast cancer patients were significantly higher than the healthy diet group at day 7 and day 2, potentially due to a decreased breakdown of circulating cells [24].

Two of these studies looked at the adherence to STF, both yielding high compliance with the respective protocols. The dose escalation from 24 h, 48 h and 72 h all had high compliance, with the highest being five out of six participants completing the 48 h cycle [25]. Riedinger et al. (2020) found that 95% of the six chemotherapy cycles were completed with strict adherence to fasting for 24 h before and after chemotherapy [27].

In sum, STF studies suggested potential benefits in reducing treatment-related toxicity and improving tolerance to chemotherapy; however, these findings were largely based on small, heterogeneous studies with varying fasting durations and timing relative to treatment. Differences in protocol implementation limit direct comparability across studies and underscore the need for more standardized approaches [24,25,27] [Table 10].

### 3.7. IF Interventions

Out of the twenty studies, two implemented IF interventions. An RCT of an 18 h IF the day before, during and after chemotherapy for 2 months (n = 24) compared to a non-fasting group (n = 24) in HER2-negative breast cancer patients conducted by Omar et al. (2022) [23] assessed toxicity and hematologic and metabolic parameters. There were no significant differences between groups in the incidence of grade I and II toxicity, but 50% of the control group experienced mouth sores and diarrhea compared to the IF group [23]. There was a significant decrease in red blood cell count and hemoglobin count with an increase in platelet count after four cycles of chemotherapy for the fasting and the control group [23]. However, there were no significant differences in hematologic profiles between both groups at cycle 4 of chemotherapy [23]. Additionally, there was also a significant decrease in the median insulin level in the IF group compared to the control group between the two time points of measurement [23].

Klement et al. (2023) [37] conducted a feasibility pilot study of a 5:2 IF prior to radiation (n = 12) compared to a standard diet (n = 12) in patients with non-metastatic breast cancer receiving adjuvant radiotherapy [37]. This study also assessed aspects of the metabolic profile but found the only statistically significant change to be gamma-glutamyl transferase (GGT) levels in the fasting group [37]. This study investigated changes in body weight, with the fasting group losing more weight at the end of the intervention compared to the control (2.45 ± 1.19 kg vs. 0.80 ± 1.21 kg) [37]. This study was the only IF intervention to assess acceptability, with 42% of patients reporting feeling “good” or “very good” during the fasting day and 58% rating the fasting schedule as easy to follow [37].

In sum, evidence for IF interventions remains limited, with few studies directly evaluating clinical or metabolic outcomes in cancer populations. Existing studies vary substantially in design and outcome reporting, making it difficult to draw consistent conclusions regarding efficacy or feasibility [23,37] [Table 11].

### 3.8. Fasting + Altered Diet Interventions

Overall, six studies investigated a form of fasting in combination with an altered diet, such as a Mediterranean, Atkins or ketogenic diet (KD) [26,28,29,30,31,40]. Of these, two studies looked at STF or mSTF, one in combination with a Mediterranean diet [26] and one KD [29]. Both studies assessed acceptability and AEs [25,29], with one also assessing toxicity, metabolic and hematologic parameters [29]. Bauersfeld et al. (2018) [26] conducted a 4-month RCT of a 60 h STF cycle (n = 27) compared to an NC Mediterranean diet (n = 23) among patients with breast or ovarian cancer undergoing chemotherapy. AEs like headache, hunger, and nausea were rated as mild during the first fasting cycle but were not attributable to the intervention [26]. Additionally, FACIT-F scores had a more significant reduction after the second half of chemotherapy and fasting cycles in the fasting group (24.8 ± 13.7) compared to the control (31.7 ± 12.6) [26]. Acceptability was also rated well, with 82% of participants rating the effectiveness of mSTF as “very good” and 91% would fast again during chemotherapy [26]. Zorn et al. (2020) [29] conducted a 5.5-month four-arm controlled cross-over pilot investigating the impact of a 96 h STF on an NC diet (n = 11), an NC diet on a 96 h STF (n = 16), a KD with a 96 h STF on an NC diet (n = 4) and an NC on a KD with a 96 h STF (n = 20) in breast, endometrial, ovarian and cervical cancer patients receiving neoadjuvant, adjuvant or palliative chemotherapy. Even with a longer fasting period than the latter, the AEs when fasting were low-grade, in addition to reported toxicities of grade I/II [29]. Notably, toxicities can cause chemotherapy postponements, which were significantly reduced during mSTF cycles [29]. In terms of acceptability, 23 participants rated mSTF as “very good”, and over 50% would fast again during chemotherapy [29]. Zorn et al. (2020) [29] noted reductions in mean corpuscular volume, hemoglobin and blood sodium as well as IGF-1 levels during mSTF cycles.

Three of the studies followed IF protocols in conjunction with a low-carb, high-protein diet like Atkins or KD [28,31,40], with two of the studies enrolling participants with glioma undergoing chemotherapy [31] or radiation therapy [29]. Schreck et al. (2021) [31] conducted an 8-week single-arm phase II study of a 5:2 IF, 5 days of Atkins and 2 days fasting, measuring adherence, change in metabolic parameters and AEs. About 72% of participants adhered to both Atkins and fasting with one day ≥ 40 g of carbohydrates, and fasting was well tolerated, with 12 participants reporting grade II nausea, diarrhea and fatigue [31]. Hemoglobin A1c and insulin levels decreased from baseline to the end of study, with a −0.2 ± 0.2 and −0.4 ± 0.6 change, respectively [31]. Voss et al. (2020) [28] implemented a randomized trial of 3-day interval cycling of a calorically restricted KD and IF fasting (n = 25) compared to a balanced diet (n = 25). The study found that the fasting group lost more body weight (2.1 ± 1.8 kg) compared to the control (0.7 ± 1.4 kg) at the end of the intervention period as well as a significant decrease in the glucose levels of the fasting group: −11.2 ± 16 mg/dL on day 6 compared to the control group with 1.4 ± 11.2 mgdL [28].

Lughmani et al. (2025) [40] investigated the impact of a 23:1 IF combined with a KD (n = 15), compared with a routine diet (n = 15) and a 23:1 IF with a routine diet (n = 15) on adenosine monophosphate-activated protein kinase (AMPK) levels and BMI in breast cancer patients receiving chemotherapy. Findings indicate both IF groups showed an average BMI reduction of 4.17 kg/m^2^, while the control group had no change [40]. Analysis also revealed a significant increase in AMPK levels in the IF-KD group from 4.66 to 10.46, indicating heightened response compared to the IF-RD group, with an increase from 4.84 to 7.37 [40]. Additionally, the control group showed a non-significant decrease from 5.58 to 5.13 [40].

The final study conducted by Lugtenberg et al. (2021) [30] assessed the impact of a 4-day FMD combined with a plant-based diet (n = 66) starting the day of each chemotherapy cycle compared to a routine diet (n = 65) on QOL and illness perceptions. QOL scores were similar in both the FMD (80.5) and control group (79.5) at baseline, deteriorated during chemotherapy in both the FMD (70.3) and control group (71.2) and then improved again in both the FMD (75.8) and control group (78.70) at follow-up [30]. Compared to the control group, the FMD group had more positive perceptions of illness, understanding, consequences and observed identity by the follow-up period [30]. These findings were numerically but not statistically significant [30]. Lastly, adherence was investigated, with 81.5% of participants completing the first FMD cycle and 21.5% adhering to all FMD cycle [30].

In sum, studies combining fasting with altered dietary interventions demonstrated generally favorable feasibility and safety profiles, with several studies reporting improvements in metabolic parameters such as glucose regulation, insulin levels, and BMI. However, findings were heterogeneous and should be interpreted in the context of study design and sample size, as many studies were small, single-arm, or pilot trials with varying levels of methodological rigor. While some randomized and controlled studies suggested potential benefits in metabolic outcomes and treatment-related toxicity, others reported only modest or non-significant differences compared to control groups. Additionally, the diversity in dietary approaches, including KD, Atkins, Mediterranean, and plant-based diets, combined with varying fasting protocols, limits direct comparability across studies. These findings suggest that while combining fasting with dietary modification may enhance metabolic responses and patient acceptability, the relative contribution of each component remains unclear. Further well-designed RCTs with standardized intervention protocols and larger sample sizes are needed to better understand the synergistic effects of fasting and dietary strategies in oncologic populations [26,28,29,30,31,40] [Table 12].

### 3.9. Cross-Cateogory Comparison

When comparing across fasting intervention categories, several key patterns emerge. FMD and STF protocols demonstrated more consistent effects on metabolic biomarkers and treatment-related outcomes, including reductions in IGF-1 and improvements in chemotherapy tolerance, although these findings were largely derived from small pilot or feasibility studies [17,24,25,27,32,34,35,39]. In contrast, TRE interventions showed consistently high adherence and feasibility but more modest and variable effects on metabolic and clinical outcomes, with fewer high-strength trials evaluating these endpoints [33,36,38,41]. IF studies were limited in number and heterogeneous in design, making it difficult to draw consistent conclusions regarding efficacy [23,37]. Studies combining fasting with altered dietary approaches, such as KD, Mediterranean, or plant-based diets, suggested potential additive metabolic effects; however, the diversity in intervention design and outcome reporting limits the ability to distinguish the independent versus synergistic contributions of fasting and dietary modification [26,28,29,30,31,40]. Across all categories, variability in study design, sample size, and methodological rigor contributed to heterogeneity in findings, highlighting the need for more standardized and well-powered trials.

## 4. Discussion

This review assessed five different categories of fasting protocols across 20 studies and their subsequent outcomes for patients with cancer undergoing treatment or in survivorship. Most of these studies were rated with moderate strength due to several factors. Some of these studies followed a pilot design, which on our scale was rated as moderate. Pilot studies are preparatory investigations designed to test feasibility, acceptability and operational readiness rather than providing evidence of effectiveness [42]. While essential to the field of research, this impacts the strength of evidence presented in the review. Additionally, some of these pilot studies were also single-arm, weakening the evidence presented. With the absence of a control group, it is difficult to attribute observed effects to the intervention itself; however, it is understood that it may be difficult to have a control group in certain high-risk populations [43]. Due to the nature of the designs, the study samples were on the small side and limit the generalizability of the findings to a larger population. Larger-scale RCTs are warranted in this area of research to strengthen the evidence.

Six studies looked at the impact of different fasting protocols on chemotherapy treatment-induced toxicity with FMD, IF, mSTF and mSTF +KD finding improvements in reported toxicity [17,23,24,25,29]. FMD [17], IF [23] and mSTF [24,27] showed positive trends towards improved gastrointestinal (GI) tract toxicities, with reduced IGF-1 and glucose levels, indicating the DSR may be working to protect the GI tract from treatment-induced destruction and toxicity. However, studies have shown that longer fasting periods are required to cause major changes in IGF-1 levels to sustain DSR [44]. An FMD protocol of 4 days every 21 days found less grade I/II GI toxicities [17], as well as an 18 h IF protocol finding similar results in the reported toxicities without a significant change in IGF-1 levels [23]. However, glucose levels, which can promote DSR when they are reduced in a fasting state, were elevated in the non-fasting group compared to the IF group [23]. Similarly, two mSTF protocols of 48 h garnered results of decreased GI toxicities in the fasting group compared to the control [24,27], although, when comparing the IGF-1 levels between FMD cohorts of 24 h, 48 h and 72 h, the changes were similar between the 24 h and 72 h cohort [25]. The authors note this may reflect non-compliance to a greater degree in cohorts with longer fasting durations [25]. However, the cycling of a 96 h fast and KD found IGF-1 levels to be significantly decreased during the fasting cycles, using ketone body monitoring as a measure of fasting compliance in addition to a food diary [29]. Together, these findings not only highlight the role of fasting duration on toxicities but also how methods of compliance monitoring can be used to improve outcomes for cancer patients within fasting studies. Additionally, these findings relate only to chemotherapy, primarily for breast cancer and gynecological cancers, and further investigation is needed for other cancer types and treatments, such as radiation.

In that regard, this review includes research that is largely focused on chemotherapy and female reproductive cancers. These are important considerations for fasting-based interventions, especially as they pertain to weight loss as an outcome. For example, populations such as patients with ovarian cancer have a significant risk for malnutrition prior to and during treatment, making calorie restriction or fasting difficult to implement [27]. Exploring how fasting-based interventions with less strict caloric restriction can impact the outcomes of cancer patients, including overweight or obese patients, is vital. Only one study in this review assessed a TRE diet in obese or overweight breast cancer patients receiving radiation and found success with weight loss from baseline [41]. It is also important to consider how other treatment types can lead to weight gain. Prostate cancer patients receiving androgen deprivation therapy (ADT) can gain weight from treatment and are at risk for increased mortality and toxicities [35]. One study in this review shows that an FMD with a plant-based diet can be used in prostate cancer patients with elevated body weight without negatively altering their nutritional status [35]. Additionally, two studies in this review assessed patients with glioma receiving radiation and partaking in an IF in conjunction with a modified Atkins diet or KD [28,31]. Higher body weight and high levels of metabolic biomarkers, both associated with a poor prognosis, were reduced in patients receiving the intervention [28,31]. Efforts should be made to expand research in overweight cancer patients or in populations that may be at risk for weight gain through treatment. Concerns of caloric restriction and malnutrition may be mitigated through more flexible fasting interventions like IF and TRE or incorporate specialized diets to complement treatment goals (i.e., keto, Atkins).

In terms of feasibility and acceptability, TRE interventions had the highest feasibility and acceptability among the interventions, with between 90 and 100% adherence to the eating window across the interventions [33,36,38,41]. Additionally, participants planned to continue the diet and would recommend it to their peers. This aligns with emerging research that TRE is more adherent than forms of calorie restriction, such as FMD [45]. TRE is easy to operate with high flexibility based on work schedules and routines without changing one’s original dietary preferences. FMD studies that are paired with specialized diets, such as plant-based [30] or Mediterranean [26], experienced participant drop-out due to aversion to the prescribed diet. It is important to note that three of the four TRE studies were in cancer survivors, who have different circumstances than those in active treatment [33,36,38]. Drop-out due to aversion to prescribed diet in FMD interventions could potentially be attributed to treatment side effects [26,30]. Further research should explore how TRE can be tolerated and adhered to in other cancer populations with treatment considerations.

When interpreted alongside the quality metric assessment, several important distinctions emerge across fasting intervention categories. While FMD and STF protocols appeared to demonstrate more consistent effects on metabolic and treatment-related outcomes, these findings were often derived from studies of moderate methodological strength, frequently limited by small sample sizes and feasibility-focused designs [17,24,25,27,32,34,35,39]. In contrast, TRE interventions were consistently associated with high adherence and acceptability across studies, including those with relatively stronger study designs; however, the observed metabolic and clinical effects were generally more modest and variable [33,36,38,41]. This pattern suggests that while TRE may represent a more feasible and sustainable approach for patients, it may not provide a sufficient physiological stimulus to achieve the magnitude of metabolic or treatment-related benefits observed in more intensive fasting protocols. Importantly, more intensive interventions, such as FMD, STF, or fasting combined with dietary modification, may offer greater biological impact but are inherently more demanding and may require additional support to ensure adherence and safety. As such, future studies should not only evaluate the efficacy of these interventions but also focus on optimizing implementation strategies, including the integration of digital health tools, remote monitoring, and structured health coaching to support patient engagement. Emerging approaches such as continuous glucose monitoring (CGM) and app-based dietary tracking may provide real-time feedback and improve adherence to more-intensive fasting protocols. Collectively, these findings highlight the need to balance intervention intensity with feasibility and to develop scalable strategies that enable patients to safely engage in interventions that are both effective and sustainable in real-world oncology settings. Future research should prioritize well-designed, adequately powered RCTs with longer-term follow-up to evaluate sustained clinical and metabolic outcomes, establish more standardized and reproducible fasting protocols, and systematically examine how fasting strategies can be optimally integrated with different dietary approaches to maximize therapeutic benefit while maintaining feasibility and patient-centered implementation.

## 5. Limitations

This review presents promising evidence for the potential benefits of fasting in cancer treatment and survivorship. Several limitations should be noted when interpreting the outcomes and proposed future directions in this review. There is significant heterogeneity between studies, including age, race and ethnicity, measures, duration, sample size, treatment trajectory and protocol. This scoping review may lack generalizability due to a large variation in sample sizes, no studies including information on socio-economic status or education, and limited studies including the race and ethnicity of their sample. The lack of information about the study sample characteristics makes comparison between studies difficult. Particularly with diet and food-related behavior modifications, socioecological factors play a key role in individual perception, acceptability and overall readiness to adhere. Additionally, the age range across the studies is wide, but more research needs to be done to assess the efficacy of fasting interventions in younger patients with cancer. While most studies primarily recruited patients with breast and/or gynecological cancers, there is a lack of homogeneity in the cancer type being assessed. This may limit the generalizability of the analysis as treatment, outcomes and toxicities may vary by not only cancer type, but treatment type and frequency. It is inconclusive if fasting interventions have an impact on illness perceptions as only one study assessed this measurement with no significant differences.

## 6. Conclusions

This scoping review mapped the current clinical evidence on fasting-based dietary interventions among individuals with cancer and survivors, encompassing a diverse range of study designs, cancer populations, intervention approaches, and reported outcomes. The findings highlight important variability across intervention types, with more intensive approaches such as fasting-mimicking diets and short-term fasting appearing to demonstrate more consistent metabolic and treatment-related effects, whereas time-restricted eating shows higher feasibility and adherence but more modest and variable clinical impact. Overall, fasting-based interventions appear to be generally feasible and well tolerated in selected populations; however, the evidence remains heterogeneous and is largely derived from studies of moderate methodological strength. As such, these findings should be interpreted with caution. Future research should prioritize well-designed, adequately powered randomized controlled trials that include diverse cancer populations, standardized and reproducible fasting protocols, and longer-term follow-up to evaluate sustained clinical outcomes. In addition, further work is needed to determine how fasting strategies can be optimally integrated with different dietary approaches and supported through novel engagement strategies, including digital health tools, remote monitoring, and behavioral support, to enhance adherence, scalability, and real-world implementation.

## Figures and Tables

**Table 1:** IF regimens definitions.

Intervention	Definition
IF	Voluntary alternating periods of eating and fasting
FMD	Structured, low-calorie diet typically followed for 5 days with repetition of the diet every few weeks
TRE	Limiting daily caloric intake to a consistent window aligned with circadian rhythms, typically 4–12 h
STF or mSTF	Typically involves water-only fasting period lasting more than 24 h up to 72 h with personalized modifications

**Table 2:** Sample size.

Score	Criteria
0	<100 participants
1	110–500 participants
2	500+ participants

**Table 3:** Study design.

Score	Criteria
0	Cross-sectional or open label
1	Prospective cohort or single-arm pilot
2	Randomized controlled trial (RCT)

**Table 4:** Intervention characterization and protocol clarity.

Score	Criteria
0	Intervention poorly described (fasting duration, schedule or diet not clearly defined)
1	Intervention defined but limited details on implementation or adherence monitoring
2	Detailed protocol including fasting schedule, duration, dietary guidance, and adherence monitoring

**Table 5:** Outcome measurement and reporting quality.

Score	Criteria
0	Outcomes are poorly defined, descriptive or inconsistently reported. Measurement tools, timing of assessment and analytic methods are unclear.
1	Outcomes are defined but limited in rigor. Studies may primarily report feasibility indicators (e.g., adherence, acceptability, or adverse events) or clinical outcomes measured using non-standardized or partially described methods.
2	Outcomes are clearly defined and measured using validated instruments or objective clinical metrics. Examples include standardized fatigue or quality-of-life instruments (e.g., FACIT-F, FACT-G), toxicity grading systems (e.g., CTCAE), or laboratory biomarkers (e.g., IGF-1, insulin, hematologic markers). Feasibility outcomes (e.g., adherence rates, acceptability, safety monitoring) are also clearly reported when relevant

**Table 6:** Analytical rigor and risk-of-bias considerations.

Score	Criteria
0	Outcomes reported descriptively without clear statistical analysis or analytic framework. No comparator group or pre–post evaluation is provided, and statistical testing, uncertainty measures, or analytic methods are not described.
1	Basic statistical analysis is conducted, such as descriptive statistics, pre–post comparisons, or simple between-group comparisons. However, analyses may lack adjustment for confounders, detailed methodological description, or reporting of effect sizes or confidence intervals. Feasibility outcomes (e.g., adherence rates or acceptability) may be summarized without formal statistical testing.
2	Appropriate statistical analyses are clearly described and aligned with the study design. Studies may include randomized or controlled comparisons, longitudinal analyses, or clearly reported pre–post analyses with appropriate statistical tests. Effect estimates, significance testing, and outcome comparisons are reported transparently. When feasibility outcomes are assessed, adherence, safety, or acceptability data are systematically analyzed and clearly presented alongside clinical outcomes.

**Table 7:** Strength of evidence across included studies.

Study	Sample Size	Design	Intervention Characterization & Protocol Clarity	Outcome Measurement & Reporting Quality	Analytical Rigor	Total Score
de Groot et al. 2015 [24]	0	1	1	2	1	5
Dorff et al., 2016 [25]	0	2	2	2	2	8
Bauersfeld et al., 2018 [26]	0	1	2	2	2	7
Riedinger et al., 2020 [27]	0	2	1	1	1	5
Zorn et al., 2020 [29]	0	1	2	2	2	7
Lugtenberg et al., 2021 [30]	1	0	2	1	1	5
Schreck et al., 2021 [31]	0	1	2	2	2	7
Valdemarin et al., 2021 [32]	0	1	2	2	1	6
Kleckner et al., 2022 [33]	0	1	2	2	2	7
Fay-Watt et al., 2023 [35]	0	1	1	0	2	4
Omar et al., 2022 [23]	0	2	2	2	2	8
Vernieri et al., 2022 [34]	1	1	2	1	1	7
Voss et al., 2020 [28]	0	1	1	2	0	4
Kirkham et al., 2023 [36]	0	1	2	2	1	6
Klement et al., 2023 [37]	0	1	2	2	2	7
Bahrami et al., 2024 [17]	0	2	2	2	2	8
Vega et al., 2024 [41]	0	1	1	0	1	3
Kleckner et al., 2025 [38]	0	1	2	2	2	7
Ligorio et al., 2025 [39]	0	1	1	2	2	6
Lughmani et al., 2025 [40]	0	2	2	1	2	7

**Table 8:** Characteristics of studies implementing FMD interventions.

Author, Year	Geographical Location	Design	Sample Size	Characteristics	Cancer Type & Treatment	Duration	Intervention	Outcomes
Valdemarin et al., 2021 [32]	Italy	Single-arm, phase I/II clinical trial	N = 90	Age (mean ± SD): 50.4 ± 8. 86% female	Colorectal Prostate Glioma Melanoma Ovarian Non-small-cell lung Pancreatic Adenocarcinoma Anal cancer Cervical Endometrial Small-cell lung Bladder Multiple Myeloma Leukemia (ALL & CML) Essential thrombocytosis Polycythemia vera + Active anticancer treatment: chemotherapy, endocrine therapy, TKIs, CDK4/6 inhibitors, proteasome inhibitors, immunotherapy, radiotherapy, and biologics	6 mos	5-day FMD 4 days before chemotherapy cycle & once a month FMD for other treatments	↓ Body weight ↓ IGF-1 levels ↑ Feasible + FMD adherence AEs: Grade I and II headache
Fay-Watt et al., 2023 [35]	Ireland	Prospective pilot implementation study	N = 35	Age (Mean ± SD): 69 ± 6.9 100% male	Prostate	3 mos	FMD for 4 continuous days for 3 cycles	↓ Body weight ↓ Total cholesterol AEs: Insignificant
Vernieri et al., 2022 [34]	Italy	Single-arm prospective clinical trial	N = 101	Age (<60): 27.7% Age (>60): 72.3% 72.3% Female	Breast Colorectal Lung Prostate Pancreas Melanoma Germinal Ovary Thyroid CLL NHL Uterus Sarcoma Multiple myeloma Stomach cancer Kidney cancer Mesothelioma Myelofibrosis + Receiving different types of anti-tumor treatment	8 mos	5 day FMD every 21 to 28 days	↓ Low-density CD15+ granulocytes by day 5 ↓ Total monocytes by day 5 ↓ IGF-1 levels ↑ Feasibility + FMD adherence AEs: Grade III & IV
Bahrami et al., 2024 [17]	Iran	Randomized controlled trial	N = 44	Intervention mean age: 49.36 ± 8.19 Control mean age: 50.9 ± 8.59	HER 2-negative breast cancer + Neoadjuvant chemotherapy	5 mos	4 day FMD every 21 days for 8 chemotherapy cycles	Toxicity: Grade III ↑ Erythrocyte count ↑ Neutrophil count ↓ IGF-1 levels
Ligorio et al., 2025 [39]	Italy	Pilot randomized phase II clinical trial	N = 30	Median age: 50.2 years (IQR: 46.9–56.2)	Triple-negative breast cancer (naïve, unilateral, stage I–III,) + Neoadjuvant chemotherapy	5 mos	5 day FMD every 21 days for 8 chemotherapy cycles	↓ BMI in overweight or obese patients ↓ IGF-1 levels ↓ Blood glucose levels ↓ Lactate dehydrogenase ↑ FMD adherence AEs: Severe (3%)

Symbols: ↑, increased value; ↓, decreased value.

**Table 9:** Characteristics of studies implementing TRE intervention.

Author, Year	Geographical Location	Design	Sample Size	Characteristics	Cancer Type & Treatment	Duration	Intervention	Outcomes
Kleckner et al., 2022 [33]	United States	Prospective single-arm pilot	N = 39	Age (mean ± SD): 61.5 ± 12.5 Female = 92.3%. Black, Asian, Hispanic and mostly White	Breast (majority), prostate, and uterine cancer survivors + Adjuvant chemotherapy, surgery and/or radiation	2 wks	14 days of a 14:10 TRE	↑ FACIT-F score from baseline ↓ Body weight ↑ Feasibility ↑ TRE adherence
Kirkham et al., 2023 [36]	Canada	Single-arm feasibility study	N = 22	Age (mean ± SD): 66 ± 5 Aboriginal, Asian, other and mostly White BMI > 25 kg/m^2^	Breast cancer survivors + Completed anthracycline chemotherapy	8 wks	8-week ad libitum 16:8 TRE eating from 12 to 8PM on weekdays	↓ BMI ↑ Feasibility + TRE adherence
Vega et al., 2024 [41]	Chile	Prospective interventional non-randomized clinical trial	N = 21	Mean age: 52	Breast cancer + Radiotherapy	12 wks	12 weeks of a 16:8 TRE	↓ Body weight ↓ Glucose levels ↑ Feasibility + TRE adherence
Kleckner et al., 2025 [38]	United States	Pilot randomized controlled trial	N = 30	Mean age: 54.1 ± 14.7 years. 76.7% female. 53.3% Black/African American, 43.3% White, other, and mixed race. 6.7% Hispanic	Breast cancer survivors + Post-treatment (2 mos–2 yrs)	12 wks	10 h daily eating window	↑ FACIT-F score ↑ Body weight ↑ Feasible + TRE adherence

Symbols: ↑, increased value; ↓, decreased value.

**Table 10:** Characteristics of studies implementing STF or mSTF interventions.

Author, Year	Geographical Location	Design	Sample Size	Characteristics	Cancer Type & Treatment	Duration	Intervention	Outcomes
de Groot et al., 2015 [24]	Netherlands	Randomized 1:1 ratio pilot trial	Intervention: n = 7 Control: n = 6	Intervention median age: 51 (47–64) Control median age: 52 (44–69)	Stage 2 & 3 HER2-negative breast cancer + Neoadjuvant or adjuvant chemotherapy	4 mos	48 h fast every 21 days for 6 cycles of chemotherapy	Toxicity: Grade I/II ↑ Erythrocyte count ↓ IGF-1 levels
Dorff et al., 2016 [25]	United States	Phase I, open-label, non-randomized trial	N = 20	Median age 61 (31–75) 75% female, 15% Male Hispanic, Black, Asian and mostly White	Urothelial, ovarian, uterine and breast cancer + Platinum-based chemotherapy	2 cycles of treatment	Fast for either 24 h, 48 h and 72 h for 2 planned chemotherapy cycles	Toxicity: Grade I/II ↓ IGF-1 levels ↓ Insulin levels ↑ STF adherence
Riedinger et al., 2020 [27]	United States	Randomized controlled trial	Intervention: n = 10 Control: n = 10	Intervention mean age: 59.5 + 10.33 Control mean age: 59 + 10.23	Ovarian, uterine and cervical cancer + Chemotherapy	4 mos	48 h fast every 21 days for 6 cycles of chemotherapy	Toxicity: Grade I/II ↑ Hematologic profile ↑ STF adherence

Symbols: ↑, increased value; ↓, decreased value.

**Table 11:** Characteristics of studies implementing IF interventions.

Author, Year	Geographical Location	Design	Sample Size	Characteristics	Cancer Type & Treatment	Duration	Intervention	Outcomes
Omar et al., 2022 [23]	Egypt	Randomized controlled trial	Intervention: n = 24 Control: n = 24	Intervention age (mean ± SD): 43.96 ± 7.37 Control age (mean ± SD) 47.42 ± 11.73	HER2-negative breast cancer + Chemotherapy	2 mos	18 h IF the day before, during and after chemotherapy every 21 days for 4 cycles of chemotherapy	Toxicity: Grade I/II RBC count Hemoglobin count ↑ Platelet count Insulin levels
Klement et al., 2023 [37]	Germany	Feasibility pilot study	Intervention: n = 12 Control: n = 12	Intervention mean age: 51.8 ± 6.9 Control mean age: 45.8 ± 10.6	Non-metastatic breast cancer + Adjuvant radiotherapy post conservation surgery	1.5 mos	5:2 IF 2 days prior to first radiation session	Body weight GGT levels ↑ Acceptability

Symbol: ↑, increased value.

**Table 12:** Characteristics of studies implementing fasting + altered diet interventions.

Author, Year	Geographical Location	Design	Sample Size	Characteristics	Cancer Type & Treatment	Duration	Intervention	Outcomes
Bauersfeld et al., 2018 [26]	Germany	Randomized cross-over pilot trial	N = 34	Age (mean ± SD) = 51.6 ± 8.4, Range: 28–69	Breast or ovarian cancer + Chemotherapy	4 mos	60 h STF cycle for 6 cycles of chemotherapy every 21 days + Mediterranean diet	↓ Fatigue ↑ Acceptability AEs: Mild
Zorn et al., 2020 [29]	Germany	4-arm controlled cross-over pilot	N = 51	Age (mean ± SD): 54 ± 11	Breast, endometrial, ovarian and cervical cancer + Neoadjuvant, adjuvant or palliative chemotherapy	5.5 mos	96 h mSTF to normo-caloric diet to ketogenic diet	Toxicity: Grade I/II ↓ Mean corpuscular volume ↓Mean hemoglobin ↓ Mean blood sodium ↑ Acceptability ↓ IGF-1 level AEs: Low-grade
Lugtenberg et al., 2021 [30]	Netherlands	Multicenter, open-label, phase II randomized study	N = 129	Intervention median age: 49 (31–71) Control median age: 51 (27–71)	HER2-negative breast cancer + Neoadjuvant chemotherapy	6 mos	FMD + Plant-based diet prior to/day of chemotherapy	↑ QOL ↑ Illness perceptions ↑ FMD adherence
Schreck et al., 2021 [31]	United States	Single-arm phase II study	N = 25	Age (mean ± SD): 50.1 (12.7) 48% women	Glioma + Adjuvant chemotherapy	8 wks	5:2 IF + modified Atkins	↓ Hemoglobin A1c ↓ Insulin level ↑ IF & Atkins adherence AEs: Grade II
Voss et al., 2020 [28]	Germany	Prospective randomized trial	N = 50	Intervention median age: 56 (39–71) Control median age: 58 (26–75)	Recurrent malignant glioma + Radiation therapy	6 mos	3-day IF, followed by 3-day calorie restriction, follow by 3-day ketogenic diet	↓ Body weight ↓ Glucose level
Lughmani et al., 2025 [40]	Pakistan	Non-randomized clinical trial	N = 45	Age (mean + SD) Group 1: 45.2 ± 5.6 Group 2: 46.8 ± 6.1 Group 3: 47.3 ± 5.9	Breast cancer + Chemotherapy	4 wks	23:1 IF with ketogenic diet	↓ Body weight ↑ AMPK level

Symbols: ↑, increased value; ↓, decreased value.

## Data Availability

The original contributions presented in this study are included in the article/Supplementary Material. Further inquiries can be directed to the corresponding author.

## Supplementary Materials

The following supporting information can be downloaded at: https://www.mdpi.com/article/10.3390/nu18071035/s1, File S1: PRISMA-ScR Checklist.

## Abbreviations

The following abbreviations are used in this manuscript:

NCD	Non-communicable diseases
DALYS	Disability-adjusted life years
IF	Intermittent fasting
FMD	Fasting-mimicking diet
TRE	Time-restricted eating
STF or mSTF	Short-term fasting or modified short-term fasting
IGF-1	Insulin-like growth factor-1
DSR	Differential stress resistance
VEGF	Vascular endothelial growth factor
QOL	Quality of life
RCT	Randomized controlled trial
NC	Normo-caloric
FACIT-F	Functional assessment of chronic illness therapy-fatigue
GGT	Gamma-glutamyl transferase
KD	Ketogenic diet
AMPK	AMP-activated protein kinase
BMI	Body mass index
AEs	Adverse events
ADT	Androgen deprivation therapy
